# A Review of Bacteria-Animal Lateral Gene Transfer May Inform Our Understanding of Diseases like Cancer

**DOI:** 10.1371/journal.pgen.1003877

**Published:** 2013-10-17

**Authors:** Kelly M. Robinson, Karsten B. Sieber, Julie C. Dunning Hotopp

**Affiliations:** 1 Institute for Genome Sciences, University of Maryland School of Medicine, Baltimore, Maryland, United States of America; 2 Department of Microbiology and Immunology, University of Maryland School of Medicine, Baltimore, Maryland, United States of America; 3 Greenebaum Cancer Center, University of Maryland School of Medicine, Baltimore, Maryand, United States of America; Université Paris Descartes, INSERM U1001, France

## Abstract

Lateral gene transfer (LGT) from bacteria to animals occurs more frequently than was appreciated prior to the advent of genome sequencing. In 2007, LGT from bacterial *Wolbachia* endosymbionts was detected in ∼33% of the sequenced arthropod genomes using a bioinformatic approach. Today, *Wolbachia*/host LGT is thought to be widespread and many other cases of bacteria-animal LGT have been described. In insects, LGT may be more frequently associated with endosymbionts that colonize germ cells and germ stem cells, like *Wolbachia* endosymbionts. We speculate that LGT may occur from bacteria to a wide variety of eukaryotes, but only becomes vertically inherited when it occurs in germ cells. As such, LGT may happen routinely in somatic cells but never become inherited or fixed in the population. Lack of inheritance of such mutations greatly decreases our ability to detect them. In this review, we propose that such noninherited bacterial DNA integration into chromosomes in human somatic cells could induce mutations leading to cancer or autoimmune diseases in a manner analogous to mobile elements and viral integrations.

## Introduction

Many eukaryotic chromosomes contain DNA of microbial origin that arose via lateral gene transfer (LGT). One such example is the directed transfer of *Agrobacterium tumefaciens* DNA that results in crown gall disease in plants and that has been used to create transgenic crops. *A. tumefaciens* specifically transfers 10–30 kbp of DNA from its 200–800 kbp tumor-inducing (Ti) plasmid to plants via the bacterial type IV secretion system [1]. The DNA from the Ti plasmid (T-DNA) is targeted to the nucleus, incorporated into the plant chromosome by illegitimate recombination, and transcribed from eukaryotic promoters in the T-DNA [2], [3]. LGT events are not limited to occurring between microbes and plants, but can also occur between microbes and animals. In this review, we synthesize our current understanding of the potential for LGT from bacteria to the somatic human genome by examining (a) LGT in animals with a particular emphasis on bacteria-animal LGT, (b) insertional mutagenesis in the human genome, and (c) the role of microbes in oncogenesis. Current and future work is then presented through two hypotheses about such integrations and their potential role in bacteria-associated chronic human diseases like cancer. Such transfers in the human genome may have been missed previously because they would not be inherited, and until recently, most LGT research has focused solely on the inherited consensus genome (e.g., [4]–[6]). However, important mutations are not limited to merely the vertically inherited genome. For example, several recent studies (e.g., [7]–[10]) have shown that the somatic genome can carry important novel mutations related to disease.

## LGT in Animals

### An Overview of LGT in Animals

Since plant germ cells are not physically protected, *A. tumefaciens*–mediated LGT in plants may be expected to occur more frequently when compared to inherited LGT in vertebrate animals where germ cells are protected. Yet, LGT can be detected in many animals, including vertebrates [11], [12]. For example, phylogenetically related antifreeze proteins in fish are scattered across disparate fish taxa, indicating a role for lateral gene transfer [13]. These proteins allow fish to survive at temperatures below freezing by preventing the formation of ice crystals [13]. Pea aphids and the two-spotted spider mite have both been found to synthesize carotenoids from an LGT that may have originated from fungi [14], [15]. In aphids, the resulting red-green color polymorphism changes the insect's susceptibility to natural enemies [14], while in the spider mite these carotenoid biosynthetic genes are differentially expressed in diapause [15], the arthropod equivalent to hibernation.

### Overview of Functional Bacteria-Animal LGT

In addition to the eukaryote-eukaryote transfers described above, several functional LGTs have been described between bacteria and invertebrate animals. For example, in mealybugs, LGTs from at least three different bacterial lineages have resulted in hybrid biosynthetic pathways [16]. These pathways are composed of genes of eukaryotic ancestry in the mealybug genome, genes of bacterial ancestry in the mealybug genome, and genes of bacterial ancestry in the obligate endosymbiont bacterial genome [16]. For example, riboflavin biosynthesis requires one endosymbiont genome–encoded protein and three insect genome–encoded proteins that arose via LGT that have α-Proteobacteria and Bacteroidetes ancestry [16]. The bacterial donors of these bacteria-eukaryote LGTs are proposed to be facultative bacterial symbionts, not the primary obligate endosymbionts [16].

Bacteria-animal LGTs have also been observed in multiple agricultural pests where the LGT facilitates parasitization of an agricultural crop. For example, *Hypothenemus hampei*, the coffee berry borer, acquired a *Bacillus* mannanase gene, which allowed it to exploit coffee berries as a new ecological niche relative to the insect's sister taxa [17]. Several plant parasitic nematodes have acquired plant cell wall–degrading enzymes from bacteria that allow the nematodes to invade plant tissues and exploit plants as a new ecological niche. More specifically, LGT followed by duplication resulted in 60 genes of bacterial origin in the nematode *Meloidogyne incongita* genome, including cellulases, pectate lyases, and expansin-like proteins that degrade or modify plant cell walls and are not typically found in other animals [18]. These proteins have been biochemically characterized, are secreted into plant tissues, and are involved in parasitism [18]. *M. incongita* also acquired cellulase genes via an additional independent LGT by *Pristionchus* nematodes [19], [20], necromenic nematodes that live in association with beetles.

### A Case Study: *Wolbachia* Endosymbionts Have Recently Transferred DNA to Multiple Invertebrate Host Genomes

In contrast to the examples above, where single genes were transferred, are functional, and have persisted over time, *Wolbachia*-insect LGTs span many hundreds of kilobases of DNA, have no evidence of being functional with no obvious change in insect phenotype, and are likely recent given that they have minimal divergence from the likely donor. Of the systems being studied today, recent LGT from bacteria to animals seems to be most prevalent between *Wolbachia* endosymbionts and their invertebrate hosts. In this case, the term gene is used loosely since a phenotype has not been ascribed to the transferred DNA. *Wolbachia* endosymbionts colonize a wide range of arthropods and filarial nematodes, including 20–70% of insect species, and are maternally inherited through the egg cytoplasm [21], [22]. Transmission through the egg cytoplasm provides ample opportunity for *Wolbachia* DNA to transfer to the host genome [11]. In addition, *Wolbachia* endosymbionts can sometimes colonize their host's germ stem cell [23]. Increased prevalence of LGT may be expected in hosts with colonized germ stem cells since a transfer in the germ stem cell will be inherited by a larger number of progeny in comparison with a transfer in a single gamete, like an ovum or spermatozoon.

Recent LGT from *Wolbachia* endosymbionts to their hosts has been characterized in diverse invertebrate hosts, including beetles [24]–[26], fruit flies [27], wasps [27], [28], tsetse flies [29], and filarial nematodes [27], [30], [31], and has been reviewed recently [11], [12]. In 2007, most of the genome sequencing projects (8/11) containing *Wolbachia* endosymbiont sequences showed evidence of having recent LGT between the endosymbiont genome and the host chromosome [27]. We successfully characterized LGT in all five of the hosts that we examined [27]. Therefore, we estimated that ∼70% of sequenced *Wolbachia*-infected hosts may have at least one *Wolbachia*-host LGT [27].

The high prevalence of *Wolbachia*-host LGT is not reflective of all microbe-host relationships. Many characteristics of their association have an effect on the frequency of LGT between the two organisms. One of the major factors limiting the occurrence of LGT is the proximity of the two organisms [32], which helps explain the increased prevalence of LGT between obligate intracellular endosymbionts and their hosts [11]. However, other factors are also likely at work, as evidenced in aphids where functional LGTs are not detected that arise from *Buchnera aphidicola*, the primary obligate endosymbionts of aphids [33], [34]. However, in aphids, functional LGT is detected from relatives of *Wolbachia* endosymbionts [33], [34]. Likewise, in the mealybug, LGT is attributed to facultative symbionts instead of the primary obligate endosymbionts [16]. This may suggest that LGT from *Wolbachia* endosymbionts and other similar bacteria (e.g., bacteria in the *Arsenophonus* and *Cardinium* clades) is more abundant for other reasons. For example, with *Wolbachia* endosymbionts, it may be because they do not merely pass through the germ cell, but rather colonize the germ cell and the germ stem cell. However, the characterization of transfers from *Wolbachia* endosymbionts to their hosts highlights that LGT is an ongoing process in at least some animals and that LGT can result in DNA transfers that are not functional and may never become functional.

### An Open Question: Does Somatic LGT from Bacteria Occur in Humans?

Despite extensive microbe-animal LGT in invertebrates, bacterial LGT to humans and other mammals has rarely been described. One barrier to inherited LGT in humans is the segregation of gametes. Unlike in insects and plants, the human germ line is both physically and immunologically well protected from bacteria. Nevertheless, there are 10× more bacterial cells than human cells in the human body [35]. Therefore, somatic human cells can be bathed in bacteria and have ample opportunity to be mutagenized by bacterial DNA through LGT. Such LGT will not become inherited by offspring of the human, but may be propagated through the individual's lifetime if the cell is capable of undergoing clonal expansion. LGT to somatic tissues acting as a mutagen may therefore be important in bacteria-associated diseases like cancer, chronic inflammatory diseases, and autoimmune disease. Under this scenario, somatic LGT events would not enable adaptation to a new niche, but rather have the potential to be disruptive to normal gene function. While this review focuses on the potential for integrations of bacterial DNA in the somatic human genome, it is possible that DNA may also get integrated from the food we consume.

To protect human cells from bacteria, the innate immune system uses pattern recognition receptors (PRRs) to recognize pathogen-associated molecular patterns, or PAMPs, including LPS, flagellin, lipoteichoic acid, lipoproteins, and peptidoglycan [36]. Both microbial DNA and mRNA are also PAMPs in humans [37], [38], while a subset of microbial rRNA does not elicit an immune response [38]. Therefore, we might expect that bacterial rRNA is more likely to mutagenize the human somatic genome. This is a particularly interesting proposition considering some mobile elements in animals have rRNA as their origin and are mobilized by the LINE-1 machinery [39], [40].

We hypothesize that bacterial rRNA may integrate into the human somatic genome and induce disease through random mutagenesis, as do other insertion-creating mutagens, like mobile elements and viruses. Should this integrated DNA mutagenize a gene by disrupting the coding region or causing deregulation, the consequences could potentially be severe. For example, such insertional mutagenesis by mobile elements and viruses has been associated with cancer.

## Insertional Mutagenesis of the Human Genome

### The Role of Mobile Elements in Cancer Progression

Since mobile elements are already known to mutagenize the human genome, including cancer genomes, they provide a useful comparison for thinking about mutagenesis via LGT. Only the non–long terminal repeat (LTR) retrotransposons, including LINE and *Alu* elements, have been shown to actively jump throughout the human genome and thus cause ongoing mutagenesis [41]. LINE-1 (L1) is the only non-LTR retrotransposon that has the machinery to move itself in the human genome, and it also mobilizes *Alu* elements [41], [42]. L1s and *Alu* elements move to new genomic locations through germ line retrotransposition [42], [43], but both elements tend to be inactive in somatic tissues [41]. However, their reactivation may contribute to tumorigenesis [44].

L1s have been implicated in both colon and lung cancer. An L1 insertion was found in the *APC* gene in colon tumor cells but not in normal cells [45]. In colorectal cancer, tumors can be identified that have been mutagenized by L1s while matched normal DNA samples lack such mutations, and some insertions disrupt genes with known cancer driver functions [46]. Nine L1 insertions were found to be only in lung cancer tumors, but not normal adjacent tissue, with 30% of tumors having between one and three new insertions [47]. However, it is not clear if somatic L1 insertions are passenger mutations, or if they are directly related to tumor formation [47].

With >1 million copies in the human genome, it is not surprising that *Alu* insertions are also related to carcinogenesis. *Alu* insertions have been reported in the *MLV12* and *MLL* genes associated with leukemia, the *BRCA1* and *BRCA2* genes associated with breast cancer, and the *MLH1* and *MSH2* genes leading to hereditary nonpolyposis colorectal cancer syndrome (HNPCC), among others [42]. The number of *Alu* and L1 integrations detected that are related to disease shows the potential importance of such integration events.

### Viral Integrations and Oncogenesis

Viruses are also known to integrate into the somatic human genome and cause mutations that are associated with cancer. In 2008, it was estimated that 15–20% of cancers worldwide were linked to infections by viruses, parasites, or bacteria [48]. The integration of human papillomavirus (HPV) is a critical event leading to HPV-associated tumorigenesis and may be an important biomarker of invasive cervical cancer [49]. In the absence of integration, the virus replicates but the cell maintains control of its own proliferation. However, cellular proliferation becomes deregulated when HPV integrates in a manner that results in the loss of E1 and E2, viral proteins required for transcriptional control and replication. Absence of these regulator proteins leads to subsequent deregulation of E6 causing downregulation of the *p53* pathway, which increases cell proliferation [50]. As many as 80–100% of cervical carcinoma tumors have an integration of HPV-16 or HPV-18 [51], [52] and these integrations are clonal within tumors [53], providing evidence that HPV acts as a carcinogen.

Hepatitis B virus (HBV) was linked to hepatocellular carcinoma (HCC) in 1981 [54], prompting its vaccine to be the first approved for cancer prevention [55]. HBV's exact role in formation of HCC is unclear despite the fact that its integrations can be found clonally in HCC tumors. Regardless, levels of HBV integration in tumor cells can predict patient survival. Individuals with >3 integrations have decreased survival compared to those with <3 integrations [10]. Seven human oncogenes and tumor suppressor genes have repeatedly been shown to contain HBV integrations, including *TERT*, *ITPR1*, *IRAK2*, *MAPK1*, *MLL2*, *MLL4*, and *CCNE1* [10], [56]–[59]. This vast number of genes disrupted by HBV illustrates the multitude of ways that HBV can contribute to HCC carcinogenesis.

### Mitochondrial Insertions and Disease

Integrations into the nuclear genome of human cells are not limited to mobile element and viral integrations. Transfers of mitochondrial DNA (mtDNA) into the nuclear genome have long been described and are called nuclear mitochondrial DNA segments (numts) [60]. There are various methods that have been suggested for how the mtDNA exits the mitochondria and enters the nucleus, including mitochondrial lysis, direct contact between the nucleus and the mitochondria, degradation of abnormal mitochondria, and mitochondrial DNA encapsulation inside the nucleus [61]. Once entrance into the nucleus has occurred, the mtDNA can integrate into the nuclear genome through nonhomologous end joining of double-strand break repair [61], [62].

While various numts have been reported, only a handful are implicated in disease. In one case, a reciprocal translocation occurred between chromosomes 9 and 11 resulting in a 41-bp insertion of mtDNA linking the breakpoint of chromosome 9 to the translocated portion of chromosome 11 [63]. There has been one report of a numt from the mitochondrial *coxIII* gene inserted in the *c-myc* gene of HeLa cells forming a chimeric RNA, but it is unknown if this numt contributed to carcinogenesis [64]. Other examples that link numts to human diseases are a 251-bp numt insertion into the human plasma factor VII gene causing severe factor VII deficiency [65], a 72-bp numt insertion in the *GLI3* gene associated with Pallister-Hall syndrome [66], a 93-bp insertion into *MCOLN1* related to mucolipidosis IV [67], and a 36-bp insertion into the *USH1C* gene implicated in Usher syndrome type IC [68], [69]. Thus far, most research on numts is focused on inherited mutations. While it has been noted that numts confound analyses of the mitochondrial genome in cancer samples [70], we are not aware of a published analysis of numts in the human somatic genome or cancer genomes, although it would be informative. An increase in somatic numts has been associated with aging in rats [71], and increases in both somatic numts and nuclear plastid–derived DNA, or nupts, have been associated with environmental stress in plants [72].

## The Role of Microbes in Oncogenesis

### Microbial Involvement in Tumorigenesis

Many bacterial associations with cancer have been characterized, including *Helicobacter pylori* with gastric carcinoma and gastric mucosa–associated lymphoid tissue (MALT) lymphoma [73], *Escherichia coli* with colorectal cancer [74], *Schistosoma haematobium* with bladder carcinoma [75], *Bacteroides fragilis* with colon cancer [76], and *Fusobacterium* spp. with colorectal cancer [77]. Many of these associations have been disputed, as it is often difficult to determine if an existing microbial infection is a symptom or a cause of cancer. Additionally, a single microbial species may merely be a marker for a more complex microbiome whose rare members contribute most substantially to oncogenesis, as has been observed in other systems [78]. Despite these associations between microbes and cancer, and the association of viral and mobile element integrations with cancer, microbial DNA integration has not been described for microbe-associated cancers. Prior to the widespread use of whole genome sequencing, the large size of microbial genomes made microbial integrations more difficult to detect. For example, viral integrations can be detected with Southern blots using viral-specific probes (e.g., [79]). The relatively larger genome size and thus higher complexity of microbial genomes likely precludes identifying integrations in the same manner without knowing something about the specific insert *a priori*. Sequencing reads that resemble bacterial DNA are sometimes removed from eukaryotic genome projects, further preventing identification of legitimate bacterial sequences in eukaryotic genomes. Instead, the microbial contribution to carcinogenesis is generally thought to occur via increased inflammation leading to DNA damage and secretion of bacterial effector proteins like toxins [80], [81].

## Current and Future Directions

### Hypothesis 1: Bacterial DNA Integration into Somatic Cells Could Induce Oncogenic Mutations

**Figure 1:**
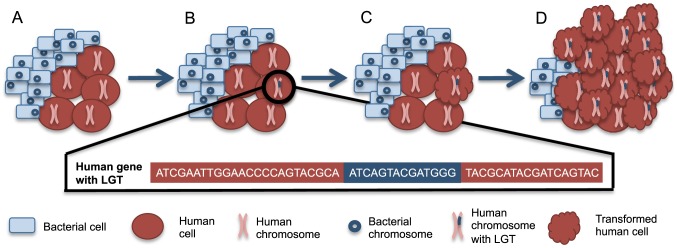
A scenario for bacteria-human LGT and carcinogenesis. (A) Normally, human cells (red circles) can coexist with a population of microbial cells (blue rectangles), which are not drawn to scale. (B) Occasionally, bacteria-human LGT may occur from one bacterial cell to one human cell as depicted by the human chromosome with bacterial DNA within it (pink chromosome with blue insertion). The bacterial sequence (blue) inserted into the human sequence (red) is illustrated below. (C) The cell with the LGT can undergo transformation to a cancerous phenotype, represented by the scalloped red cell. Such transformation may be related to the integrations or may instead be related to some other alteration in the cell. (D) The now cancerous cell clonally expands and forms a tumor where the majority of cancer cells share the original LGT.

Bacterial integration that disrupts and mutagenizes proto-oncogenes or tumor suppressor genes could provide an additional avenue for bacteria-associated oncogenesis, beyond inflammation-induced damage. Such integrations could arise through a directed mechanism, as has been observed with *Agrobacterium*-induced crown gall disease. Alternatively, these integrations may merely result from the release of nucleic acids following lysis of bacteria. Integrations could occur specifically with particular integration sites as is observed with mobile elements or randomly in a manner analogous to mutations induced by exposure to known carcinogens, like UV radiation and cigarette smoke. Because bacterial DNA and mRNA are recognized by the human immune system [37], [38], we anticipate that most bacterial integrations will arise from nucleic acids that are not recognized as PAMPs, like some rRNA [38]. Given that L1 machinery can mobilize *Alu* elements as well as cellular genes [42], we hypothesize that the L1 machinery may also play a role in integrating bacterial rRNA into the somatic human genome. Although these events could lead to any type of genetic disorder, LGT events in cancer are easier to detect due to clonal expansion within the tumor (Figure 1).

The availability of large cancer genome datasets like The Cancer Genome Atlas (TCGA) facilitates testing this hypothesis across a wide variety of cancer types. Evidence has recently been presented supporting bacterial LGTs of *Acinetobacter*-like DNA in acute myeloid leukemia samples and of *Pseudomonas*-like DNA in stomach adenocarcinoma samples [82]. There was a higher frequency of LGT in the tumor samples when compared to the available normal samples [82]. The integrations found in stomach adenocarcinoma samples were in known oncogenes and tumor suppressor genes, while the integrations in acute myeloid leukemia samples were in the mitochondrial genome or numts [82]. It was not possible with this analysis to determine if bacterial integrations contributed to carcinogenesis or occurred as passenger mutations during cancer progression. For example, cancer cells may become more permissive to mutations during carcinogenesis and thus receptive to LGT. Regardless, the clonal expansion of tumor cells containing LGTs, as depicted in Figure 1, likely facilitated this discovery. Integrations were found only from specific members of the microbiome, suggesting that LGT is limited to a subset of bacteria [82]. In these cases of mutagenesis involving explicit carcinogenic bacteria, the transfers and any resulting etiology could be prevented through the development of vaccines. Hopefully this new evidence supporting the existence of bacteria-human LGT will prompt more investigation of this topic.

### Hypothesis 2: Bacterial Integration into Somatic Cells Could Yield a Protein or Epitope

With all frequency-based interactions, even the most unlikely event can still occur, albeit very infrequently. Integration of bacterial DNA is more likely to disrupt gene function than to result in expression of transferred genes in the new host. However, following integration of bacterial DNA there is the potential for a bacterial gene to become transcribed and a protein or peptide to be synthesized. Expression of some bacterial genes, like the vitamin K biosynthetic gene, could be beneficial in intestinal cells. In contrast, the synthesis of a protein or peptide with a particular bacterial pathogenicity epitope could elicit an adverse immune reaction to a human cell. Such a reaction could in turn lead to an immune reaction to human epitopes. In combination with the well-established mechanism of imperfect removal of autoreactive lymphocytes [83], this could lead to an autoimmune response and possibly an autoimmune disease. The autoimmune response may persist for a lifetime since it is based on the human epitopes. But, the DNA integration that began the chain reaction may not persist or be detected since the human cell expressing the bacterial epitope may be destroyed in the initial immune reaction. It is important to consider that this is only a hypothesis and no data has been presented to demonstrate that this occurs. While this hypothesis may be more unlikely than the first, it is an idea that should be considered.

## Conclusions

Extensive LGT has been detected between bacteria and animals, particularly between endosymbionts and their hosts. Recent LGT may be associated specifically with endosymbionts that colonize germ cells and the germ stem cell of their respective hosts. The extensive LGT observed between *Wolbachia* endosymbionts and their invertebrate hosts suggests that LGT involving bacteria and animals may occur more frequently than was thought a decade ago. While vertebrates have an immune system and segregated gametes that may prevent transfers like those seen in invertebrates, transfers to the vertebrate somatic genome have not been appreciated and warrant further examination. Bacterial DNA integration may be a mutagen associated with noninherited genetic diseases, like cancer, as described in a recent paper demonstrating LGT from *Acinetobacter* spp. in leukemia samples and from *Pseudomonas* spp. in stomach cancer samples.
